# Bumblebee social learning outcomes correlate with their flower-facing behaviour

**DOI:** 10.1007/s10071-024-01918-x

**Published:** 2024-11-26

**Authors:** Yuyi Lu, Zhenwei Zhuo, Mark Roper, Lars Chittka, Cwyn Solvi, Fei Peng, Ying Zhou

**Affiliations:** 1 Department of Neurology, Shenzhen Hospital, Southern Medical University, Shenzhen, 518110 China; 2 Department of Psychology, School of Public Health, Southern Medical University, Guangzhou, 510515 China; 3 Guangdong-Hong Kong-Macao Greater Bay Area Center for Brain Science and Brain-Inspired Intelligence, Southern Medical University, Guangzhou, 510515 China; 4 Department of Psychiatry, Zhujiang Hospital, Southern Medical University, Guangzhou, 510282 China; 5 School of Computer Science and Electronic Engineering, University of Essex, Colchester, CO4 3SQ UK; 6 Drone Development Lab, Ben Thorns Ltd, Colchester, CO7 9PF UK; 7 Department of Psychology, School of Biological and Behavioural Sciences, Queen Mary University of London, London, E1 4NS UK

**Keywords:** Insects, Observational learning, Pose-estimation, Second-order conditioning

## Abstract

Previous studies suggest that social learning in bumblebees can occur through second-order conditioning, with conspecifics functioning as first-order reinforcers. However, the behavioural mechanisms underlying bumblebees’ acquisition of socially learned associations remain largely unexplored. Investigating these mechanisms requires detailed quantification and analysis of the observation process. Here we designed a new 2D paradigm suitable for simple top-down high-speed video recording and analysed bumblebees’ observational learning process using a deep-learning-based pose-estimation framework. Two groups of bumblebees observed live conspecifics foraging from either blue or yellow flowers during a single foraging bout, and were subsequently tested for their socially learned colour preferences. Both groups successfully learned the colour indicated by the demonstrators and spent more time facing rewarding flowers—whether occupied by demonstrators or not—compared to non-rewarding flowers. While both groups showed a negative correlation between time spent facing non-rewarding flowers and learning outcomes, the observer bees in the blue group benefited from time spent facing occupied rewarding flowers, whereas the yellow group showed that time facing unoccupied rewarding flowers by the observer bees positively correlated with their learning outcomes. These results suggest that socially influenced colour preferences are shaped by the interplay of different types of observations rather than merely by observing a conspecific at a single colour. Together, these findings provide direct evidence of the dynamical viewing process of observer bees during social observation, opening up new opportunities for exploring the details of more complex social learning in bumblebees and other insects.

**Supplementary Information:**

The online version contains supplementary material available at 10.1007/s10071-024-01918-x.

## Introduction

Social learning refers to the acquisition of a new behaviour resulting from the observation of another animal (Heyes 1994; Zentall 2012; Carcea and Froemke 2019), which allows animals to acquire new information from others while reducing the need for individual exploration and exposure to risk (Olsson et al. 2020). In the past few decades, a mounting number of studies have demonstrated social learning in a wide range of species beyond humans, from non-human primates to insects (Leadbeater and Chittka 2007; Leadbeater and Dawson 2017; Carcea and Froemke 2019; Olsson et al. 2020).

Many experiments on social learning across species can be explained by domain-general learning mechanisms such as non-social, basic associative learning (Chittka and Leadbeater 2005; Olsson et al. 2020; Singh et al. 2021). Bumblebees have been extensively studied as insect models for such research, providing significant evidence to support this viewpoint (Olsson et al. 2020; Singh et al. 2021). In a typical bumblebee learning-by-observation paradigm (Worden and Papaj 2005; Dawson et al. 2013; Avarguès-Weber and Chittka 2014), an observer bee is allowed to enter an enclosed transparent chamber where they can see their conspecifics (live or artificial model demonstrator) foraging on coloured flowers. After a period of observation, the observer bee can develop a preference for demonstrator-occupied flowers over unoccupied flowers. Using this paradigm, it has been further demonstrated that bumblebees’ learned preferences directly reflect their previous experiences with demonstrating conspecifics and reinforcement (Dawson et al. 2013). That is, if a bee experienced finding and consuming reward, i.e. sugar water, alongside conspecifics, they later approached flowers they had observed other bumblebees visiting. Conversely, if a bee encountered aversive conditioning with conspecifics, e.g. bitter quinine solution, they subsequently avoid flowers they observe other bumblebees visiting. This implies the occurrence of second-order conditioning, a fundamental associative learning mechanism, wherein demonstrators act as first-order reinforcers.

Previous studies have focused on the outcomes of observational learning in bumblebees, rather than the detailed behavioural processes involved. However, how bumblebees perform social observation remains largely unclear. Investigating these detailed behavioural processes can offer additional support for the associative account of social learning. For example, live demonstrators may move and forage from specific flowers, one at a time, exposing observers to situations where demonstrators are physically separated from flowers, and thus requiring them to make generalisations to unoccupied but socially indicated flowers (Avarguès-Weber et al. 2015). Understanding such observational processes can shed light on the behavioural dynamics and mechanisms at play.

Here we set out to examine bumblebees’ behaviour during observational learning and asked whether their spatial positions and body orientations can account for individual learning outcomes. We first designed a new paradigm that constrained bees to move in a near 2D space, making it suitable for simple top-down high-speed video recording, as opposed to previous studies that typically allowed bees to move in 3D. We then employed a recently developed animal pose-estimation framework using deep neural networks (Mathis et al. 2018; Lauer et al. 2022) to extract the observer bees’ detailed positions and heading directions. Note that bumblebees, like all sighted insects, have compound eyes with large fields of view (Spaethe and Chittka 2003; Land and Chittka 2013; Taylor et al. 2019).

In bumblebees, this may be particularly relevant, as they have a limited frontal binocular zone, which spans only a small range of azimuth (Taylor et al. 2019), indicating that they need to orient towards stimuli to achieve effective depth perception and target detection. Indeed, hoverflies exhibit this orientation behaviour by stabilising their head or body direction to detect small, relevant objects in their environment (Land 1999). Similarly, bees tend to re-orient themselves to face behaviourally relevant stimuli directly, and their stimulus-specific brain activities have been shown to precede and predict these re-orientation behaviours (Paulk et al. 2014). Therefore, we assume that the heading directions of bumblebees serve as a good indicator of their binocular vision, which may be used to detect social cues and stimuli, similar to flower-facing behaviour observed in previous research (Frasnelli et al. 2021). With this assumption, we set out to explore the specific cues the observer bees spent time viewing during observation and how these cues affected their learned colour preferences.

## Methods

### Animals and setup

**Fig. 1:**
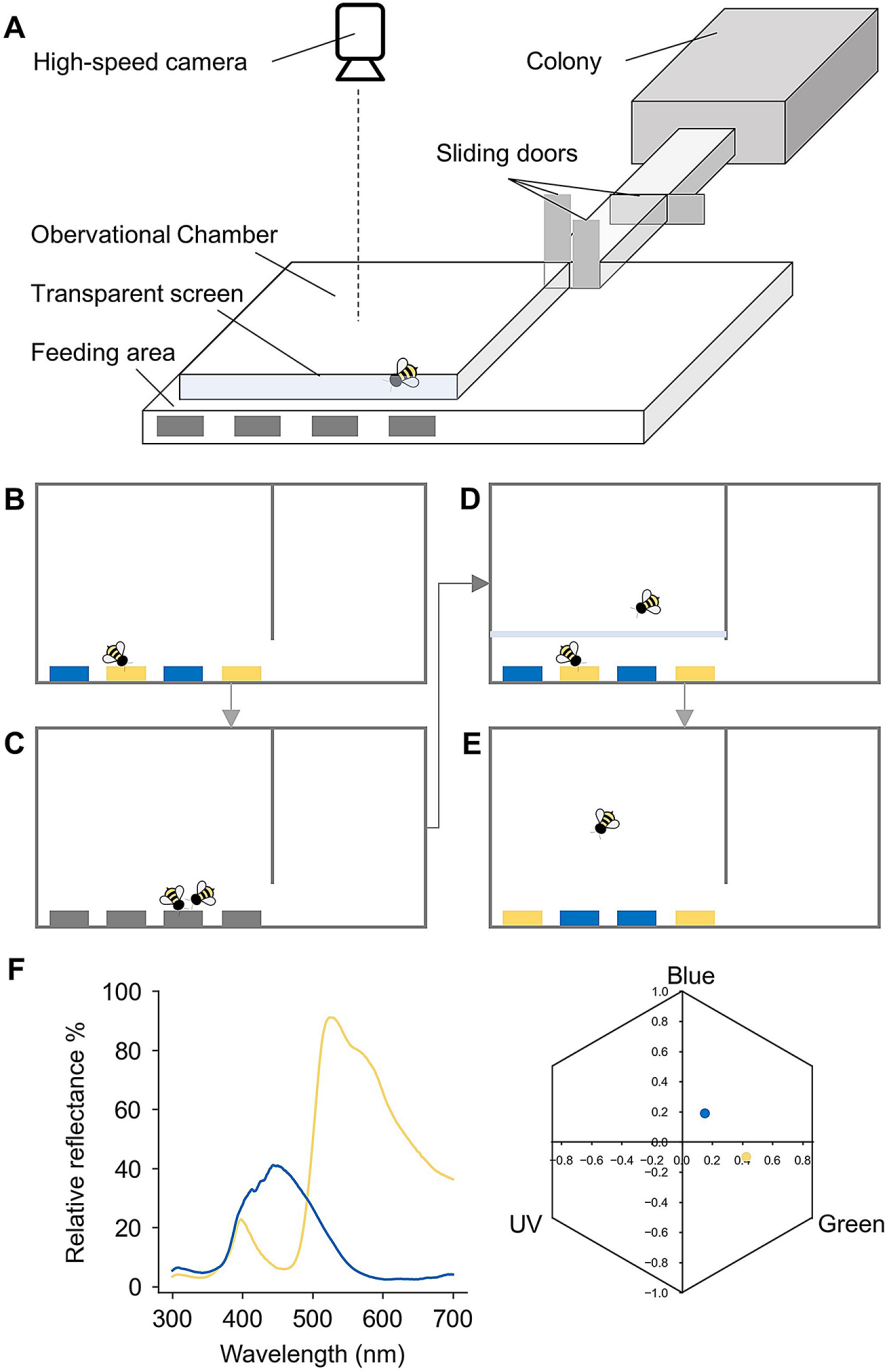
The bumblebee observational learning setup and experimental procedures. (**A**) The experimental apparatus. The foraging arena was separated into an observational chamber and a feeding area where four artificial flowers were mounted to the back wall of the arena. (**B**) Demonstrator bees were individually pre-trained to forage on one type of flower (blue or yellow). (**C**) Demonstrator bees and naïve observer bees were paired to co-feed on grey flowers for three consecutive bouts. (**D**) In the observation phase, observer bees were then individually released into the observational chamber for the duration of one bout of demonstrator feeding. (**E**) The observer bees were then individually given access to non-rewarding flowers for 5 min to determine their socially-learned flower preferences. (**F**) Spectral reflectance of the two colours used in the experiments, and the loci of colours in the hexagonal bee colour space, determined by the bees’ UV, blue and green photoreceptors (Chittka 1992)

Two bumblebee (*Bombus terrestris*) colonies from the Chinese branch of Biobest (Biobest Biotechnology, Shouguang, China) were housed in wooden nest boxes (28 cm × 16 cm × 11 cm) and were fed daily with 20% (w/w) sucrose solution and pollen ad libitum in their nest boxes outside of experiments. An acrylic tunnel with sliding doors was used to connect the nest boxes and a foraging arena (Fig. 1A), permitting experimental control of individual bees’ access to the arena. Illumination was provided by two simulated daylight LED tubes (TruD65TM, 3nh, Shenzhen, China). Each individual bee was marked with a number tag (Opalithplättchen, Warnholz & Bienenvoigt, Ellerau, Germany) attached to her thorax with superglue.

Bumblebee experiments were carried out within a customised foraging arena (25.6 cm × 12.8 cm × 2.1 cm; Fig. 1A) that contained a transparent observational chamber (15.2 cm × 10 cm × 2.1 cm) and a feeding area (15.2 cm × 2.8 cm × 2.1 cm), separated by a thin acrylic screen (15.5 cm × 2.1 cm × 2 mm). This transparent screen allowed the observers to only receive visual information from the feeding area while blocking olfactory cues. A high-speed camera (ILCE-9, Sony, Tokyo, Japan) was mounted above the UV-transparent acrylic ceiling of the arena. The limited height of this arena (2.1 cm; Fig. 1A) was chosen to simplify the behavioural tracking task: instead of full three-dimensional tracking with multiple cameras, bumblebees’ movements in this arena were approximately two-dimensional, and a top-down view camera sufficed in recording bumblebees’ detailed positions. Depending on the training and testing phases, four grey or two yellow and two blue artificial flowers (2.4 cm × 1.9 cm × 0.8 cm; henceforth “flowers”) were fixed to the back wall of the arena, with a separation of 1.6 cm in between each flower.

### Experimental procedures

All bees were individually pre-trained to collect sucrose solution from grey-coloured artificial flowers (bees could extend their probosces through a small hole at the centre of each flower and collect the sucrose droplets provided). We randomly selected demonstrator bees, with two assigned to the Blue group and two to the Yellow group. Each demonstrator was individually trained to find reward at one of two types of flowers (either yellow- or blue-coloured flowers containing 10 µl 20% w/w sucrose solution as reward or 10 µl saturated quinine hemisulphate solution as punishment; Fig. 1B). In each bout of foraging, a demonstrator bee could visit multiple flowers as the experimenter would refill a depleted flower from the back wall of the arena. The demonstrator training phase was completed when a bee made at least 100 flower visits, at which point each demonstrator performed at nearly 100% correct choices. Note that during the social observation phase, across all 42 sessions (42 observers in total), the demonstrators made 100% correct choices in 40 of those sessions. In the remaining two sessions, their choices were also accurate, achieving 11 out of 12 correct choices in one session and 12 out of 13 in the other (demonstrators made on average of 11.333 ± 2.270 visits to the flowers). Naïve or observer bees were then individually paired with colour-trained demonstrators to co-feed on grey flowers (Fig. 1C) for three consecutive bouts, to ensure the observer had formed a first-order, positive (reward) association with the demonstrator (Dawson et al. 2013).

Two counterbalanced groups of bees underwent the observational training procedure. The Yellow group consisted of observer bees (*N* = 20) that were given the opportunity to observe demonstrators foraging on yellow flowers, and the Blue group comprised observer bees (*N* = 22) that could observe demonstrators foraging on blue flowers. During the observation, a demonstrator bee was released into the feeding area to forage from flowers of one colour (yellow or blue), while an observer bee was allowed to view the floral array and demonstrator without direct contact with them (Fig. 1D). The observation period was terminated when the demonstrator bee completed one bout of foraging and returned to the tunnel leading to the hive. Note that the one-demonstrator, one-bout observation procedure used here contrasts with the classical bumblebee social learning paradigm (Worden and Papaj 2005; Dawson et al. 2013; Avarguès-Weber and Chittka 2014), where observer bees were given a 10-min observation period to observe multiple live or model demonstrators. A naïve group of bees (Control group, *N* = 22) was also trained with the same pre-training procedure as the two experimental groups, but did not participate in any social foraging or an observation phase prior to the test phase.

The two experimental groups and the control group were then tested individually with a colour preference test (Fig. 1E). Specifically, each bee in the experimental groups was let back into the tunnel after the observation phase to wait while the entire foraging arena was replaced with one that was clean, with a different flower spatial arrangement (randomised for different bees), and with each flower filled with 10 µl of water. The observer bee was then released back into the arena (Fig. 1E) and their floral choices within 5 min were recorded. A floral choice was defined as any time a bee extended her proboscis and touched the feeding hole at the centre of a flower.

### Pose estimation and statistical analyses

To estimate the poses of the observer bees from the high-speed (100 fps) video footage taken during the observation phase, DeepLabCut (Mathis et al. 2018; Nath et al. 2019; Lauer et al. 2022) toolbox and custom code in Python (version 3.8) were used to extract coordinates of both the observer and demonstrator bees’ head, thorax and abdomen. Instances where bees were standing, walking, or flying across the floor of the arena were included in the analyses, whereas instances in which bees were hanging on the walls or ceiling were manually excluded (on average 15% of frames). After the manual inspection, estimated body part coordinates with a ‘likelihood’ (DeepLabCut output) of less than 0.99 were then further excluded. The likelihood values indicate the confidence of each body part’s position estimate, and frames with low likelihoods suggest uncertain or poorly predicted positions (Mathis et al. 2018; Nath et al. 2019; Lauer et al. 2022). Applying this 0.99 likelihood threshold led to the exclusion of less than 0.1% of additional frames, highlighting the high quality of the tracking data. We calculated the locations of bees using DeepLabCut estimated body thorax positions, and their heading angles using both estimated thorax and head positions. We then calculated the observer bees’ body orientation and categorised instances where they faced specific flowers. This was done by drawing a line from the bees’ positions along their body orientations to see if the line intersected with the location covered by specific flowers, which resulted in the following four different types of flower-facing behaviour: (1) a rewarding flower occupied by a demonstrator bee, (2) an unoccupied rewarding flower, (3) an *occupied* non-rewarding flower, where the observer occasionally saw a demonstrator in front of a non-rewarding flower due to passing or rare errors, and (4) an *unoccupied* non-rewarding flower.

Statistical analyses were performed in R (version 4.4.0) with glmmTMB (version 1.1.9) and emmeans (version 1.10.1) packages. For each group, we compared the test results to chance level using generalised linear mixed models (GLMM), using a binomial distribution and a logit link function. The dependent variable was the proportion of choices (choosing one type of flower out of two types), with the number of choices included as a weight to account for the varying choice counts among bees, and bee and colony identities were included as potential random factors. The significance of factors were tested using likelihood ratio tests (LRTs). The overall performance of the unrewarded colour preference test among different groups was also compared using binomial GLMM. The dependent variable was the proportion of choices for each bee, fitted with a binomial distribution and a logit link function, with the number of choices as a weight.The experimental group was included as a fixed factor, while bee and colony identities were included as potential random factors. LRTs were performed on nested models to examine the overall group effects, followed by post-hoc pairwise comparisons.

The first choice made by each bee among different groups was then compared using Fisher–Freeman–Halton test. The time spent facing different flowers between the two experimental groups was initially compared using nested GLMMs using LRTs. After summing the time spent facing occupied and unoccupied rewarding flowers, we compared the time difference between facing socially rewarding and non-rewarding flowers using a GLMM, assuming a Gaussian distribution. In addition, we evaluated the potential contribution of different types of observations to the learning outcomes. Specifically, the overall test performance was included as the dependent variable assuming a binomial distribution. The time spent by observer bees facing occupied rewarding flowers (when the demonstrator bee was feeding on the rewarding flower), unoccupied rewarding flowers (when the demonstrator was absent), ‘occupied’ non-rewarding flowers and ‘unoccupied’ non-rewarding flowers were included as potential fixed factors, with the colony and bee identity included as potential random factors, and the signficancy of different factors were examined using LRTs. The Blue and Yellow group were modelled separately to account for the potential innate colour preferences.

### Ethical note

Although there are no current legal requirements regarding insect care and use in research in any country, experimental design and procedures were guided by the 3Rs principles (Russell and Burch 1959; Fischer et al. 2023). The behavioural tests were non-invasive, and the types of manipulations used are similar to those experienced by bumblebees during their natural foraging life. The bumblebees were cared for on a daily basis by trained and competent staff, which included routine monitoring of welfare and provision of correct and adequate food during the experimental period.

## Results

### Bumblebees rapidly learn through observation in a simplified apparatus

**Fig. 2:**
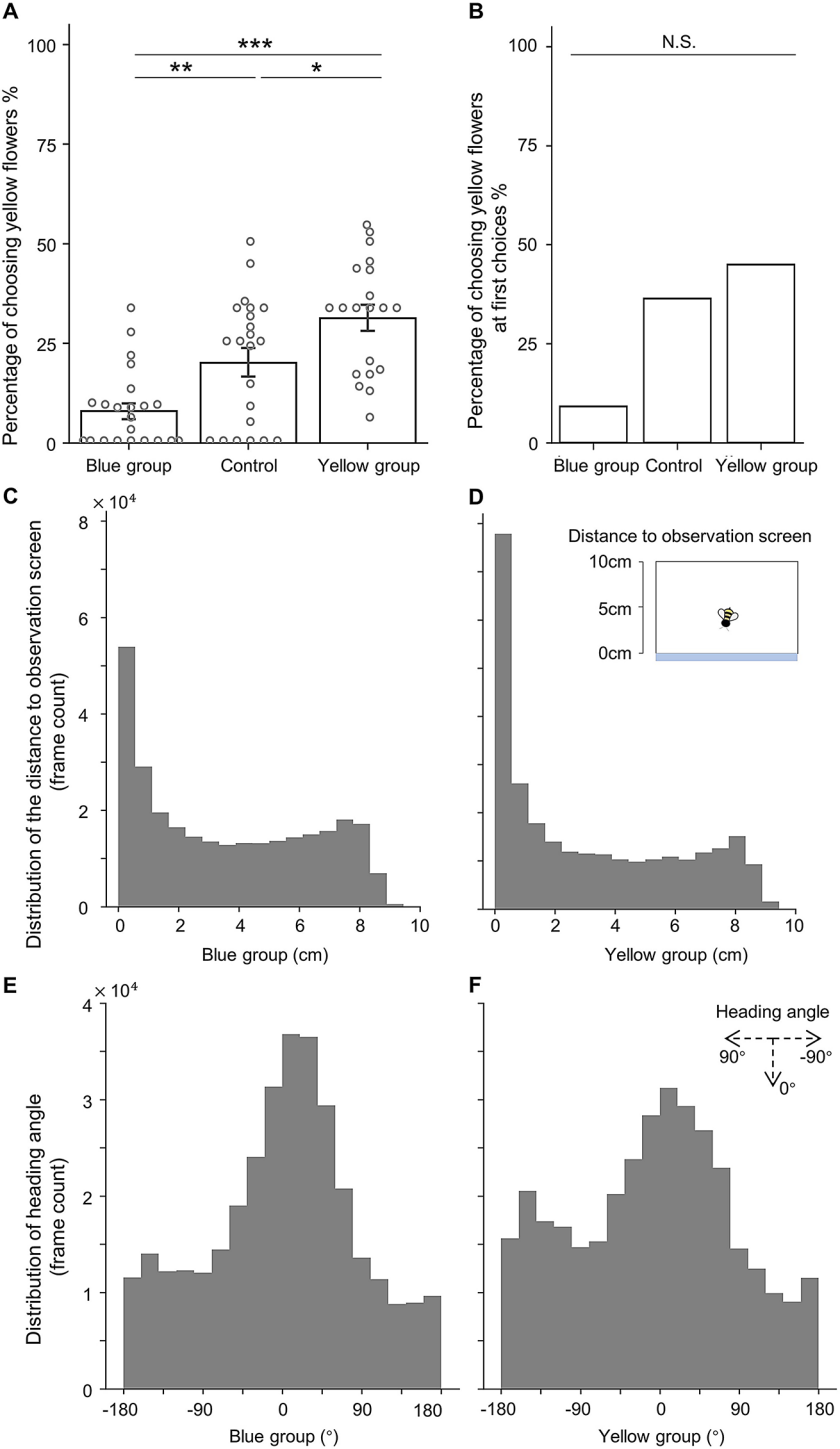
Observational learning outcomes and the positional and directional patterns of observer bees. (**A**) Preference for yellow flowers in the unrewarded test for the Blue group, Yellow group, and Control group. (**B**) First choices made in the unrewarded test across different groups. **C**. **D**. After estimating head and thorax positions for each frame during the observation phase, the distributions of the observer bees’ positional changes relative to the observational screen adjacent to the feeding area were visualised separately for the Blue and Yellow groups. **E**. **F**. Using the estimated head and thorax positions for each frame during the observation phase, the heading direction per frame was calculated, and the distributions of the observers’ heading direction changes were visualised separately for the Blue and Yellow groups. ***P < 0.001, **P < 0.01, *P < 0.05, N.S.: not significant

To verify whether bumblebees can learn colour preferences after a one-demonstrator, one-bout observation (Methods) within a customised apparatus for simplified motion-tracking (arena with restricted hight and a transparent observation chamber; Fig. 1A), we examined the bees’ choices in the subsequent unrewarded tests. The Control group revealed a clear innate bias for the colour blue (GLMM: *N* = 22, Z = − 6.009, *P* < 0.001; Fig. 2A). Similarly, both the Blue and Yellow groups also showed distinct preferences for blue, even after undergoing observational learning (GLMM for Blue group: *N* = 22, Z = − 8.404, *P* < 0.001; GLMM for Yellow group: *N* = 20, Z = − 4.715, *P* < 0.001; Fig. 2A). This aligns with findings from previous studies (Briscoe and Chittka 2001; Dawson et al. 2013; Avarguès-Weber and Chittka 2014).

Despite the preference for blue flowers, we found a significant difference in colour preference among the three groups (Likelihood-ratio test of the group effect: *P* < 0.001; Fig. 2A). Specifically, both the Blue and Yellow groups showed significantly different colour preference from the Control group, but in opposing directions (post-hoc pairwise comparison, Blue vs. Control: Z = − 3.077, *P* = 0.004; Yellow vs. Control: Z = 2.297, *P* = 0.022; Yellow vs. Blue: Z = 5.306, *P* < 0.001; Fig. 2A). Although the first choice during the test for each bee was not statistically significant (Fisher-Freeman-Halton test, *P* = 0.118, Fig. 2B), presumably due to the relatively small sample size, the overall trend was consistent with the overall test performance (Fig. 2A). These results verified that in our new paradigm customised for motion tracking, bumblebees can learn colour preferences through observation of conspecifics, despite the influence of innate colour preferences. Strikingly, the observation time in our setup was brief (ranging from 94 to 288 s; averaging 159 s), and involved only one live demonstrator, in contrast to previous studies that used 10-min observation periods and multiple live or model demonstrators (Chittka and Leadbeater 2005; Worden and Papaj 2005; Dawson et al. 2013; Avarguès-Weber and Chittka 2014). This suggests that our new paradigm is efficient in facilitating social learning in bumblebees.

### Bumblebees show distinct positional and orientational patterns during observation

To explore where bumblebees positioned themselves and oriented during social observation, we estimated the observer bees’ head and thorax positions using DeepLabCut (Mathis et al. 2018; Nath et al. 2019; Lauer et al. 2022) and calculated their heading directions (Methods). From the distributions of the bees’ thorax positions during the observation, it is visually evident that observer bees from both Blue and Yellow groups predominantly spent their time in close proximity to the observation screen (Fig. 2C, D). Moreover, the distributions of bees’ heading directions (Fig. 2E, F) reveal a tendency for observer bees to orient themselves towards the flowers on the rear wall of the arena, indicating the possibility that binocular vision may play a role in social observation. However, the fact that bees tended to spend more time near and orient towards the feeding area did not provide specific information about what they were viewing during the observation stage. We therefore analysed their heading direction in more detail.

### Bumblebees’ spend more time facing rewarding flowers than non-rewarding flowers

**Fig. 3:**
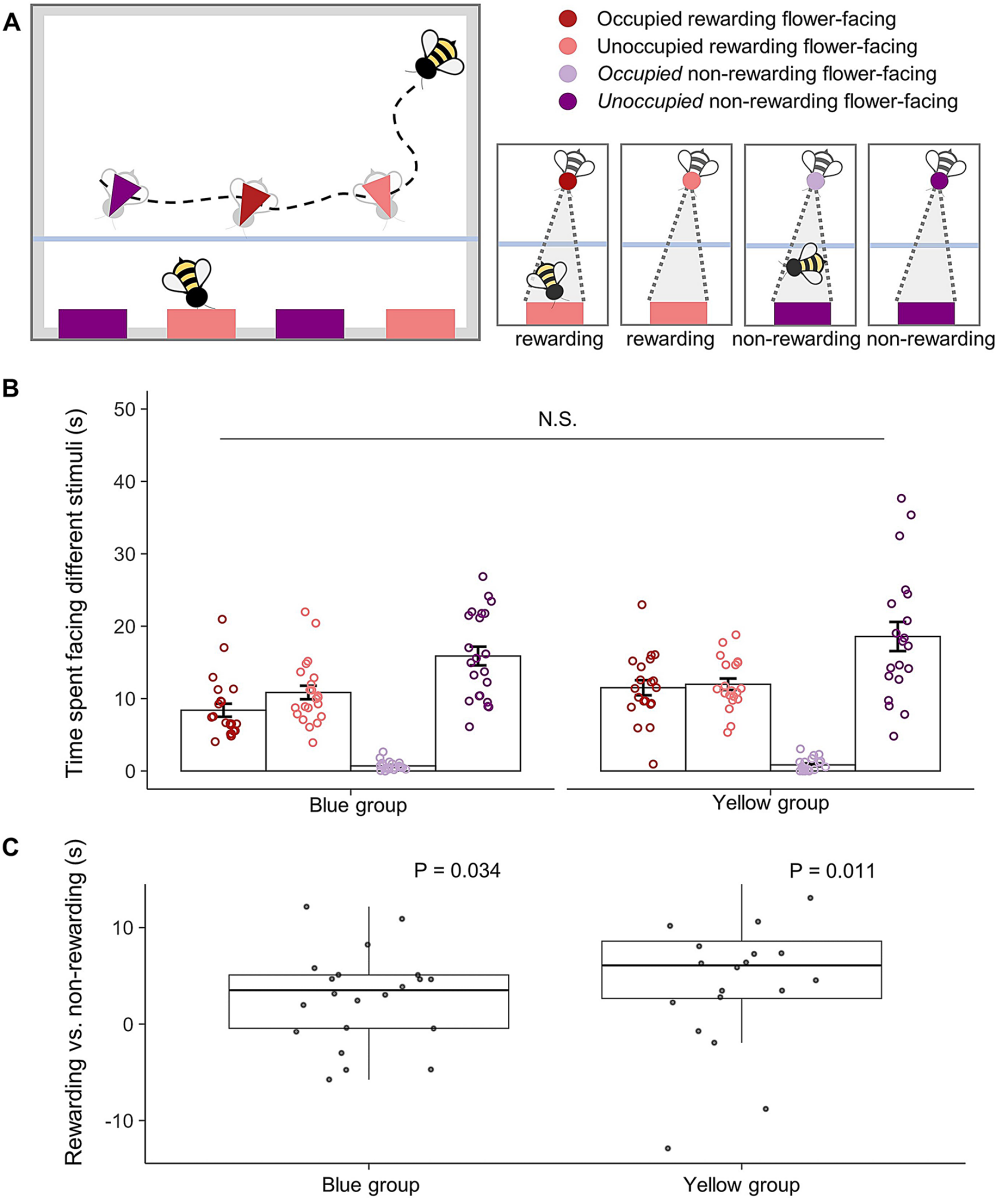
Observer bees’ time spent facing different cues. (**A**) Schematic diagram illustrating different flower-facing behaviours based on calculated heading directions and estimated positions of the observer bees. (**B**) Time spent by observer bees facing different flowers for the Yellow and Blue groups. (**C**) The relative difference in time spent facing rewarding (both occupied and unoccupied) and facing non-rewarding (*occupied* and *unoccupied*) flowers, visualised using box plots. N.S.: not significant

Based on the bees’ heading direction, the observation time was divided into periods spent facing (their body oriented towards) rewarding flowers occupied by a demonstrator, rewarding flowers without a demonstrator, non-rewarding flowers with a demonstrator and non-rewarding flowers without a demonstrator (Methods; Fig. 3A). While there was no clear difference in the time spent on each category between Blue and Yellow groups (LRT: *P* = 0.241; Fig. 3B), both groups spent significantly more time facing rewarding flowers—whether occupied by a demonstrator or not—compared to non-rewarding flowers (GLMM for the Blue group: *N* = 22, Z = 2.124, *P* = 0.034; GLMM for the Yellow group: *N* = 20, Z = 2.559, *P* = 0.011; Fig. 3C). This confirms that observing a successful forager indeed creates a strong bias in the colour preference of an observer bee.

### Correlations between flower-facing behaviours and learned colour preferences

**Fig. 4:**
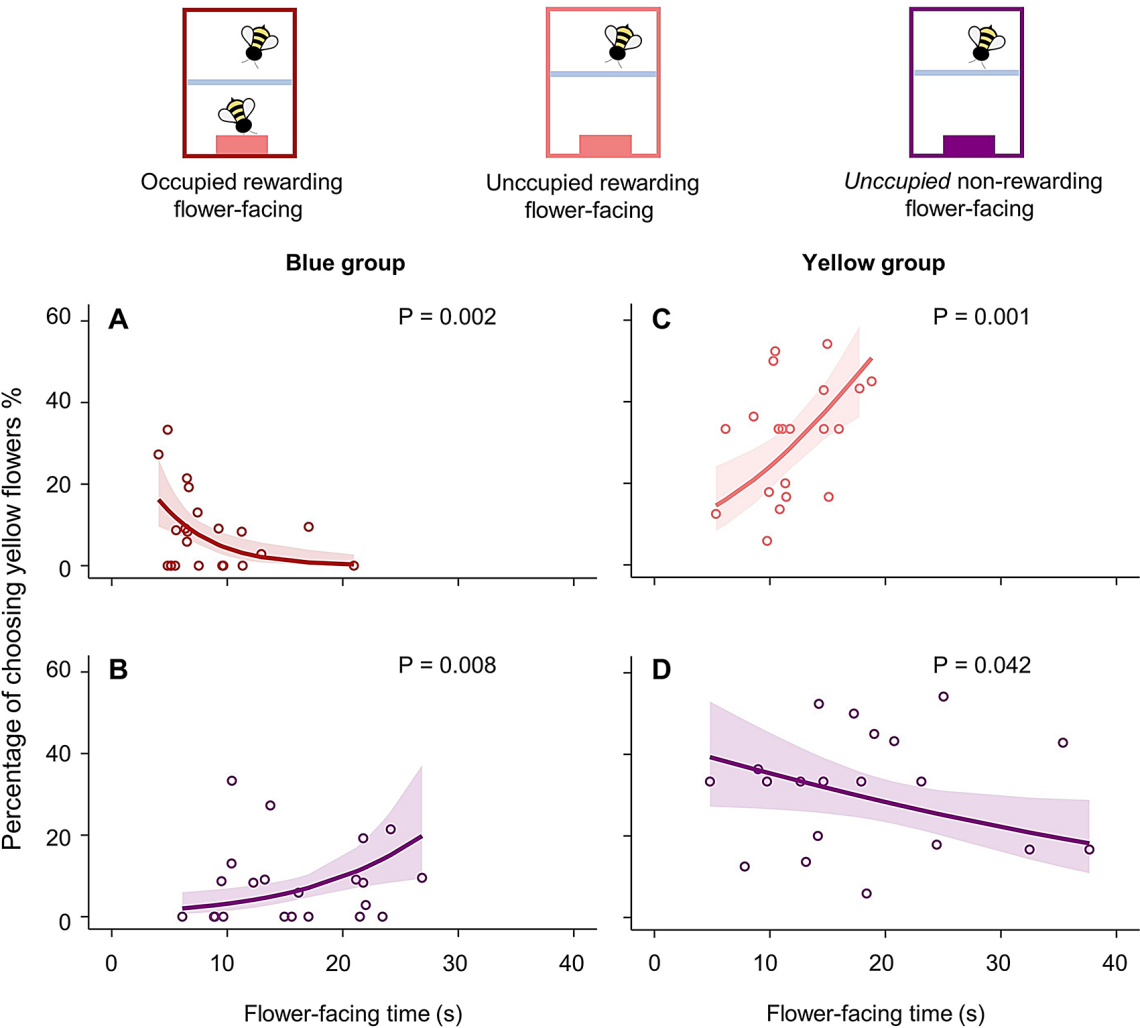
Flower-facing behaviours correlate with socially-learned colour preferences in bumblebees. **A**–**B**. The relationship between occupied rewarding flower-facing time, *unoccupied* non-rewarding flower-facing time and the test performance in the Blue group, respectively. **C**–**D**. The relationship between unoccupied rewarding, *unoccupied* non-rewarding flower-facing time and the test performance in the Yellow group, respectively

A closer examination of the correlations between different flower-facing behaviours and the bees’ learned colour preferences revealed distinct patterns. In the Blue group, the time spent facing occupied rewarding flowers positively (GLMM, *N* = 22, Z = 3.069, *P* = 0.002; Fig. 4A), and facing non-rewarding flowers negatively (GLMM, *N* = 22, Z = − 2.672, *P* = 0.008; Fig. 4B) contributed to the learned colour preference. For each additional second spent facing occupied rewarding (blue) flowers, the probability of choosing the blue flowers increased by approximately 1.50%. Conversely, for each additional second spent facing non-rewarding flowers, the probability of choosing the blue flowers decreased by approximately 0.85%.

In contrast, in the Yellow group, the time spent facing unoccupied rewarding flowers positively contributed to the test performance (GLMM, *N* = 20, Z = 3.432, *P* < 0.001; Fig. 4B), while the time spent facing non-rewarding flowers had a negative effect (GLMM, *N* = 20, Z = − 2.038, *P* = 0.042; Fig. 4C). Specifically, for each additional second spent facing unoccupied rewarding (yellow) flowers, the probability of choosing the yellow flowers during the test increased by approximately 1.61%. Conversely, for each additional second spent facing non-rewarding flowers, the probability of choosing the yellow flowers decreased by approximately 0.37%.

Could the tendency to face unoccupied rewarding flowers simply be a continuation of facing occupied rewarding flowers after the demonstrators had left? To explore this, we examined the frames immediately preceding unoccupied rewarding flower-facing behaviour. Interestingly, bees faced non-rewarding flowers more frequently than occupied rewarding flowers prior to facing unoccupied flowers (GLMM for the Blue group: *N* = 22, Z = 1.999, *P* = 0.046; GLMM for the Yellow group: *N* = 20, Z = 2.878, *P* = 0.004). These results suggest that socially-learned preferences may be driven by more than just instances of facing a demonstrator on a flower. Instead, they depend on the interplay between time spent facing different cues, such as the degree of generalisation to rewarding but unoccupied flowers (Avarguès-Weber et al. 2015).

## Discussion

Social learning has been proposed to be explained by domain-general processes, such as associative learning (Chittka and Leadbeater 2005; Heyes 2012; Heyes and Pearce 2015). A recent theoretical study further proposed that social learning outcomes correlate with the number of observation opportunities, indicating that increased exposure to social stimuli may result in a higher likelihood of response to these stimuli (Lind et al. 2019). Here we provide empirical behavioural evidence corroborating these theoretical propositions, suggesting that the outcomes of social learning in bumblebees may be a function of the frequency of observation incidences: increased observation of socially rewarding stimuli or decreased observation of non-rewarding stimuli can enhance the likelihood of bumblebees copying demonstrator’s colour choices. Our novel 2D social learning paradigm suitable for simple top-down motion tracking allowed us to verify that bumblebees can learn through observation, even with much shorter time period and fewer demonstrators, compared to previously used paradigms. Our findings begin to dissect the individual behaviours during observation that facilitate or hinder social learning.

It is not surprising that bumblebees spent most of their time nearer the flower array and most of the observation phase with their bodies oriented in the general direction of the flower patch. It was, however, not necessarily expected that bees’ body alignment with specific flowers would correlate with their socially-learned preferences. In our setup, bumblebees likely relied on their frontal binocular zone to observe live demonstrators moving and feeding among the flowers—a process that could involve depth perception, object recognition, and stimulus comparison, all of which may require binocular vision. However, whether bees can socially learn from cues located anywhere in their peripheral visual field has yet to be demonstrated experimentally. Future work will need to isolate information during social learning to determine the contribution of cues directly in front of a bumblebee’s body versus anywhere else.

Bumblebees’ capacity to gain information by observing other foragers and subsequently altering their floral preferences has been repeatedly demonstrated (Leadbeater and Chittka 2005; Worden and Papaj 2005; Dawson et al. 2013; Avarguès-Weber and Chittka 2014; Leadbeater and Dawson 2017), leading to the assumption that since observers are exposed to mobile demonstrators who are physically disassociated from rewarding flowers, the observers must generalise to the unoccupied rewarding flowers to learn the floral preferences (Avarguès-Weber et al. 2015). However, previous studies have not yet directly demonstrated how this generalisation effect develops. By quantifying the observation process, we provide empirical support showing how bees generalise to the unoccupied flowers and that the degree of generalisation correlates with their learning outcomes. It is important to note, however, that bumblebees’ innate preferences for blue over yellow colours (Briscoe and Chittka 2001; Dawson et al. 2013; Avarguès-Weber and Chittka 2014) may have obscured the impact of observation and generalisation, as results from the Blue and Yellow groups did not show the same effects. Future studies using stimuli that do not induce such innate biases could help clarify these differences.

The fact that observers can rapidly learn to change their floral choices, only spending a fraction (average 38 s) of the average 2.65 min observation period facing flowers, may have significant ecological implications. Bumblebees often forage in dense flower meadows, where dozens of flowers are likely to come into view every second (Chittka et al. 1999; Couvillon et al. 2015), providing little time for bumblebee observers to pick up pertinent social information, especially since they normally must observe while in flight themselves. Therefore, their lifestyle demands rapid transfer of values between social information and flowers.

While recent advances in computer vision and deep learning have facilitated the analysis of multi-animal social learning tasks through precise, marker-less pose estimation frameworks (Lauer et al. 2022; Pereira et al. 2022), the application of deep-learning-based behavioural analysis to insect observational learning has just begun. Such analysis could extend beyond the simple stimulus enhancement demonstrated in this study, to more complex behaviours involving sequential motor actions. For example, bumblebees have been shown to rapidly learn through observation tasks that require string-pulling (Alem et al. 2016) and ball-rolling (Loukola et al. 2017). Examining the observational processes leading to these socially-learned complex motor skills could provide insights into the mechanisms by which the miniature brains of bumblebees generate top-down expectations and activate imitative motor patterns (Wilson and Knoblich 2005).

## Electronic supplementary material

Below is the link to the electronic supplementary material.

Supplementary Material 1

Supplementary Material 2

Supplementary Material 3

## Data Availability

Data is provided within the manuscript or supplementary information files.
